# Determination of Selected Chemical Levels in Room Air and on Surfaces after the Use of Cartridge- and Tank-Based E-Vapor Products or Conventional Cigarettes

**DOI:** 10.3390/ijerph14090969

**Published:** 2017-08-28

**Authors:** Jianmin Liu, Qiwei Liang, Michael J. Oldham, Ali A. Rostami, Karl A. Wagner, I. Gene Gillman, Piyush Patel, Rebecca Savioz, Mohamadi Sarkar

**Affiliations:** 1 Center for Research and Technology, Altria Client Services LLC, 601 E. Jackson Street, Richmond, VA 23219, USA; Jianmin.liu@altria.com (J.L.); qiwei.liang@altria.com (Q.L.); Michael.J.Oldham@altria.com (M.J.O.); ali.a.rostami@altria.com (A.A.R.); Karl.a.wagner@altria.com (K.A.W.); 2 Enthalpy Analytical Inc., 800 Capitola Drive, Durham, NC 27713, USA; Gene.Gillman@enthalpy.com; 3 Inflamax Research Inc., 1310 Fewster Drive, Mississauga, ON L4W 1A4, Canada; piyush.patel@inflamax.com; 4 Clinopsis SA, Chemin des Jardins 6, 1426 Concise, Switzerland; Rebecca.savioz@clinopsis.com

**Keywords:** passive vaping, secondhand smoke, thirdhand exposure, electronic cigarettes, electronic vapor products, conventional cigarettes, room air chemicals, nicotine, propylene glycol, glycerol

## Abstract

There is an ongoing debate regarding the potential of secondhand exposure of non-users to various chemicals from use of e-vapor products (EVPs). Room air levels of 34 chemicals (nicotine, propylene glycol (PG), glycerol, 15 carbonyl chemicals, 12 volatile organic chemicals (VOCs), and four selected trace elements) were measured where EVPs and cigarettes were used by *n* = 37 healthy adult tobacco users in an exposure chamber. The products used were MarkTen^®^ 2.5% Classic (Group I), a Prototype GreenSmoke^®^ 2.4% (Group II), Ego-T^®^ Tank with subjects’ own e-liquids (Group III) and subjects’ own conventional cigarettes (Group IV). Products were used under controlled conditions and 4-h ad libitum use. Background (without subjects) and baseline levels (with subjects) were measured. Cumulative 4-h. levels of nicotine, PG and glycerol measured were several-fold below the time-weighted average limits used in workplace exposure evaluation. Most the other chemicals (>75%) were at or below the limit of quantification during EVP use. Significant levels of chemicals (17 out of 34) were observed in Group IV. Overall, our results indicate that under the study conditions with the products tested, cumulative room air levels of the selected chemicals measured over 4-h were relatively small and were several-fold below the current occupational regulatory and consensus limits.

## 1. Introduction

E-vapor products (EVPs), including both cartridge- and tank-based devices, have been gaining popularity over the last decade. Many smokers seeking alternatives to conventional cigarettes (CCs) are starting to use EVPs and some are even completely switching to these products and stopping smoking [1,2,3,4]. EVPs deliver nicotine in an aerosol that has a very different composition than a typical aerosol from CCs. There are thousands of chemicals generated from the combustion of tobacco, many of which are carcinogenic [5,6]. In contrast, far fewer chemicals are generated from heating an e-liquid consisting of carrier constituents (usually propylene glycol (PG) and/or glycerol), nicotine, water and flavors [7]. Studies have shown that the aerosols delivered by EVPs not only contain nicotine, PG and glycerol, but some EVPs may also contain certain chemicals such as carbonyls, volatile organic constituents (VOCs) and metals [8,9,10,11]. Even though these chemicals are present in EVP aerosols at much lower levels than in tobacco smoke, concerns have been raised regarding passive exposure of non-users to these chemicals from EVP use [12]. 

Scientific evidence on the potential for nonuser exposure to chemicals from EVP use is emerging. Experiments have been carried out where aerosol was generated from EVPs using smoking machines, and released in chambers of different dimensions [13,14,15,16]. Apart from e-liquid components, few, if any, additional chemicals were detected, when walk-in sized chambers were used. Only one study has reported measurable levels of VOCs (e.g., toluene and benzene) and carbonyls (e.g., acetaldehyde and formaldehyde) when the aerosol was exhaled in a bag [15]. In a recent systematic review Hess et al. [17] reported 24 full text articles related to passive vaping, of which the authors chose to include 16 articles for qualitative synthesis. The authors conclude that “Our review found that the absolute impact from passive exposure to EC vapor has the potential to lead to adverse health effects. The risk from being passively exposed to EC vapor is likely to be less than the risk from passive exposure to conventional cigarette smoke.”

Studies using smoking machines to investigate levels of potentially exhaled chemicals from EVP use are limited in that they don’t reflect real-life situations and assume that 100% of the machine generated constituents are released into the room air. Recent work by St. Helen et al. [18] measured chemical constituents in the exhaled breath of EVP users and found that on average only 6% of the nicotine, 8% of the propylene glycol, and 16% of the glycerin inhaled is exhaled. Furthermore, the aerosol profile could be misleading if aerosol generation parameters, (e.g., puff volume and puff duration) are set at conditions that do not reflect actual use behavior. For example, in a recent publication, formaldehyde precursors were detected in EVP aerosols when tested under high voltage (5.0 V) condition [19]. This method has been criticized [20] and the authors suggest that machine testing should be conducted under “realistic conditions” [21]. Nevertheless, other researchers [22,23,24,25] have reported formaldehyde levels in aerosols under machine testing conditions depending on product design, particularly for second and third generation e-vapor products. There is insufficient evidence regarding potential emission of formaldehyde in the room air where e-vapor products are used. This research study will attempt to address this gap.

Unlike CCs where secondhand smoke is also generated from the sidestream smoke due to burning of the tobacco rod between puffs, secondhand aerosol is only generated from EVP users’ exhaled breath. Although there have been reports in literature [13,14,15,16,26,27,28,29,30,31,32,33,34,35,36], the extent of potential secondhand exposure to chemicals in a room where adult EVP users are using either cartridge- or tank- based e-vapor products has not been systematically evaluated. 

Three studies report on the level of chemicals in room air when human subjects were using EVPs [13,16,35]. These studies demonstrated that EVPs could be a source of nicotine, selected VOCs, carbonyls, metals and polycyclic aromatic hydrocarbons in indoor air. However, each study had limitations, such as short sessions of EVP use under artificial use conditions [16], poorly designed baseline conditions [35], or the measurement of only a limited number of chemical constituents [13] potentially present in aerosols in rooms where precise measurements of air inflow and outflow were not carried out.

In addition to the emerging public health concerns about passive exposure to chemicals in room air when people use EVPs, there appears to be additional focus on thirdhand exposure [37]. Thirdhand exposure has been defined as exposure to EVP aerosol residue that could settle on surfaces [38]. Thirdhand exposure has been speculated to occur from the involuntary inhalation of dust or through skin contact with contaminated surfaces. Nicotine was detected on surfaces in homes of EVP users who had not used CCs for at least a year [39]. However, the level reported was not different from the traces of nicotine found in homes of non-users of nicotine-containing products. The only other study that evaluated the potential thirdhand exposure to nicotine from EVPs used a syringe to generate the vapor [40] which may not be considered representative of real-life settings.

The World Health Organization (WHO) recommended that the use of EVPs should be prohibited in indoor public places until there is enough evidence that exhaled aerosols are not harmful to by-stander [41]. However, to date, the available data on secondhand and thirdhand exposure to nicotine and chemicals from the use of EVPs is inconclusive. In addition, there appears to be significant research gaps in this area as acknowledged in literature [42]. A systematic assessment of room air levels of constituents released from use of cartridge and tank-based EVPs along with CCs would add to the body of evidence for informed decision-making.

We conducted a controlled clinical study in an exposure chamber (EC) with an integrated Heating, Ventilating, and Air Conditioning (HVAC) system where the air exchange rate, as well as the temperature was precisely controlled and measured. The objective of this study was to evaluate the release of vapor-phase nicotine, PG, glycerol and other selected chemicals (carbonyls, VOCs and metals) in exhaled breath and in room air after use of three different EVPs and CCs. Deposition of nicotine, PG and glycerol on indoor surfaces was also investigated. In this paper, we report observations from the room air and surface experiments; results from the exhaled breath experiment will be reported separately [43].

## 2. Materials and Methods

### 2.1. Study Design

All pertinent study related documents, including the study protocol, were reviewed by an Institutional Review Board of Chesapeake Institutional Review Board (Pro00010679). All study participants signed an informed consent and the study was conducted in accordance with the Declaration of Helsinki under the principles and requirements of Good Clinical Practices as defined by the Food and Drug Administration [44].

#### 2.1.1. Overall Design and Schedule (Figure 1)

The study was designed as an open-label, single center, controlled, observational study, conducted in December 2014. Thirty-seven healthy adult EVP users or adult CC smokers were enrolled. They were assigned to one of four study groups according to their product use. Eighteen cartridge- or tank-based e-vapor users were assigned to either use a commercially available e-cigarette (Group I) or a prototype e-cigarette (Group II), in a 1:1 ratio. Nine tank-based e-vapor users were assigned to use a commercially available tank system with the study participant’s own preferred e-liquid (Group III), and ten CC smokers were assigned to use their own CC brand (Group IV). Study participants underwent screening procedures following informed consent. The study participants were provided information regarding quitting tobacco products (QuitAssist^®^ brochure) and were instructed to only participate in the study if they were not interested in quitting tobacco use. Within 42 days of the screening visit, all study participants within each group came to the study site for a second visit after overnight nicotine abstinence (self-reported), for the study procedures.

All study procedures were conducted in an exposure chamber (EC, Inflamax Research Inc., Toronto, ON, Canada), set up at the research site (High Points Clinical Trials Center, High Point, NC, USA). This chamber has been validated to provide consistent environmental controls conditions and used in challenge tests used in submissions for approval of drugs by the FDA submissions [45]. The chamber has been constructed to exist as a stand-alone unit allowing for complete control of air exchange rates (fresh air supply rate of 7.5 L/s) typical of living areas in accordance with standards set by the ASHRAE (American Society of Heating, Refrigerating and Air-Conditioning Engineers) for living areas [46]. Room air samples (RAS) for the quantification of nicotine, PG, glycerol and selected chemical constituents were collected under four different conditions:

(1) *Background condition (all study days, at multiple times)*—Measurements were conducted after a complete 2-h air wash of the EC, in the absence of any study participants. The background assessment was conducted primarily to account for potential off-gassing of carbonyls and VOCs from construction material and fixtures in the EC.
(2) *Baseline condition (all Groups)*—Baseline measurements were made when study participants were sitting in the EC for four hours without using any tobacco or nicotine containing products.
(3) *Pre-specified product use condition (only Group I and II)*—Room air and surface levels were measured when study participants used the product every 30 min for four hours (each product use was pre-specified as one 5-s puff every 30 s for a total of ten puffs).
(4) *Ad libitum product use condition (all Groups)*—Room air and surface levels were measured when study participants used the product ad libitum during four hours. Groups I–III study participants were asked to take at least 40 puffs, without any puff duration or puff interval restrictions and Group IV study participants were asked to smoke at least one complete cigarette every hour. Study participants were provided a counter to track the number of puffs.

For the baseline, pre-specified and ad libitum conditions, all study participants within each group entered the EC at the same time. They were asked to remain seated and alert. No drinks or foods were allowed, except ad libitum drinking water. Exit and re-entry in the EC was limited to minimize airflow disturbance. The cartridges and tanks of all EVPs were weighed before and after use in order to assess the amount of e-liquid used.

**Figure 1:**
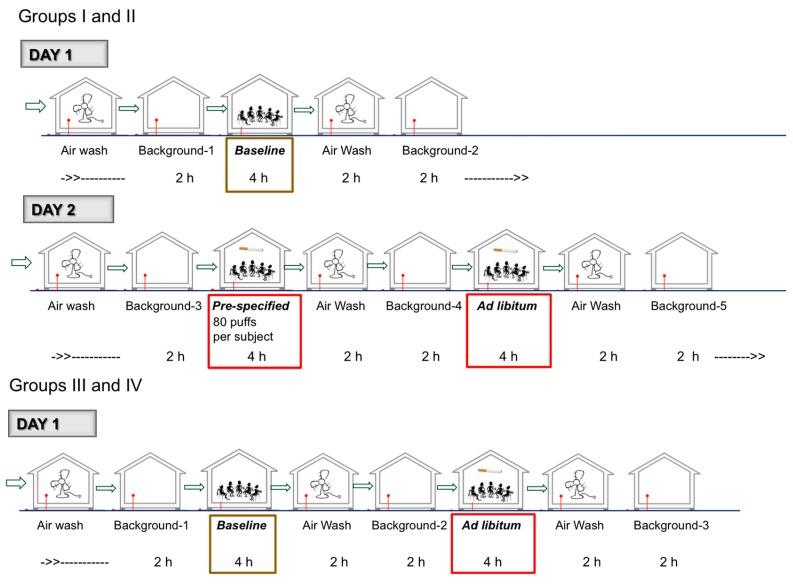
Overall Study Conduct and Sampling Schedule.

The study procedures’ schedule at the second visit is shown on Figure 1. Groups I and II study participants stayed on site for two days. Group III and IV study participants stayed on site for one day. After all procedures were completed, all study participants were given information regarding quitting tobacco products (QuitAssist^®^ brochure) and were released from the site. The study participants were not forced to use any e-vapor products or cigarettes at any point during the study.

#### 2.1.2. Study Participants

The study plan was to enroll approximately 40 male and female study participants (10 study participants per group). Study participants had to be aged 21 to 65 years, had a urine cotinine level of >200 ng/mL, a body mass index (BMI) of 18 to 40 kg/m^2^ and be in good general health as judged by the Principal Investigator (PI). Groups I and II study participants had to be users of nicotine containing EVPs, with a daily consumption of at least one cartridge EVP or at least 2 mL of e-liquid. Group III study participants had to be users of nicotine containing EVP tank products, with a daily consumption of at least 2 mL of e-liquid. Group IV study participants had to be CC smokers with a consumption of 10 to 40 predominantly non-menthol cigarettes per day (CPD). All study participants were tobacco product users for at least 30 days prior to study start.

#### 2.1.3. Study Outcome Measures

Total of 34 analytes in RAS (Table 1) and three analytes (nicotine, PG and glycerol) in surface samples were measured. The 34 analytes measured in RAS included nicotine, PG, glycerol, 15 carbonyls, 12 VOCs and four trace elements. The rationale for measuring these selected chemicals was primarily because these are the general list of chemicals typically measured for indoor air tests [8,9,10,11] and for which validated aerosol collection and analytical methods were available.

The primary outcome measures were the changes in the levels of nicotine, PG, glycerol and selected analytes in RAS from baseline (where no product was used) to product use conditions, for the four study products (background adjusted where applicable). The secondary outcome measures were the changes in the level of nicotine, PG and glycerol on surface samples from baseline to product use conditions, for the four study products.

#### 2.1.4. EC and Room Air Sampling

The EC used in this study had an area of 38 m^2^ and a volume of 114 m^3^. It was equipped with upper air return vents and lower air supply vents. Fresh air supply was set at 7.5 L/s (15 cubic feet per minute) per study participant, in accordance with standards set by the American Society of Heating, Refrigerating and Air-Conditioning Engineers (ASHRAE) for living areas [46], resulting in 2.2 air changes per hour. Room temperature was maintained at 22 °C (72 °F) ± 5 °C. The minimum and maximum temperatures recorded during the study were 20.5 °C and 23.5 °C, respectively. The average relative humidity (% RH) of the EC was 33.76 and ranged from 22.81–45.34 over the duration of the study. The EC was constructed of heavy duty polypropylene to minimize a potential sink effect for the volatile organic chemicals (VOCs). There were no additional materials, e.g., carpet or window drapes that could potentially contribute to “off-gassing” of the VOCs.

During baseline and product use conditions, RAS were collected continuously for four hours (cumulative four hour samples), counted from the time the study participants entered the EC. Background RAS were collected continuously during two hours, after each air wash consisting of at least 100 air changes. Specially designed 10-port air samplers (Enthalpy Analytical, Inc., Durham, NC, USA) were used to sample the air by continuously drawing a controlled amount of air through the sampling port using a vacuum pump. Critical flow orifices were used for each sampling port to control the flow rate. Different ports were used for the different analytes. Nicotine, PG and glycerol were collected on XAD-7 (SKC 226-95) adsorbent tubes with a target flow rate of 1000 mL/min. Particle generation was not measured in this study. We only used adsorbent tubes and specifically did not include particulate filters in our sampling to determine the chemical speciation. Carbonyls were collected on DNPH-coated silica gel adsorbent tubes (SKC 226-119) with a target flow rate of 200 mL/min. VOCs were collected on SVI thermal desorption tubes (Perkin-Elmer Inc. Waltham, MA, USA) with a target flow rate of 60 mL/min. Selected trace elements were collected on quartz filters (SKC 225-401) with a target flow rate of 1700 mL/min.

A total of four air samplers were placed in the EC at 117 cm above the floor, two of which were generally within study participants’ breathing sphere. An additional sampler (A1) was placed in the air return line outside the EC. The purpose of the sampler was to determine the levels of the chemicals in the outflowing air. The data are not presented because the levels of selected chemicals in the outflowing air were similar to the average of the room air levels measured at the different sampling stations inside the EC. We had initially included two sampling ports (A1 and A6) in the air return line, however subsequently we decided to just keep one sampling station since the second sampler yielded similar values and A6 was not analyzed to avoid the redundancy.

**Figure 2:**
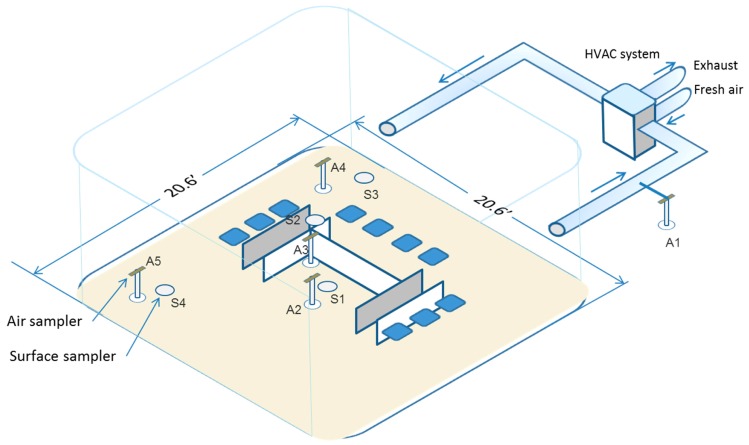
Schematic of the exposure chamber with air sampling points. HVAC: Heating, Ventilating, and Air Conditioning.

A schematic diagram of the EC showing the position of the samplers is shown in Figure 2. Prior to this study, the methods for collecting RAS and quantifying the level of nicotine, PG, glycerol, carbonyl, VOCs and metals in RAS were validated. The methodology and results of the validation study are published elsewhere [47]. Although not specifically designed to study a “proximity effect”, aerosol sampling was conducted at specified distances from users’ breathing zone in a room compliant with building codes (air changes per hour) for businesses. We have developed a computational model that should allow us to estimate the likelihood of exposure to a non-user from being in close proximity to a user of EVPs. This is a topic for a future publication.

#### 2.1.5. Surface Sampling

During baseline and product use conditions, surface samples were also collected in the EC. Four glass petri dishes (each of 15 cm diameter) at each sampling location were placed within 30 cm of each of the four air samplers, 66 cm above the floor. The dishes were uncovered immediately prior to the entry of the study participants into the EC and were collected immediately after the study participants exited. The collection media were rinsed with total of 30 mL of isopropyl alcohol, containing the appropriate internal standards. The determined mass of each compound and the surface area of dish were used to calculate mass/area (μg/cm^2^).

#### 2.1.6. Products Used

Group I study participants were assigned to use a cartridge-based EVP (marketed as MarkTen^®^ by NuMark LLC, Richmond, VA, USA). The e-liquid in the cartridges consisted of 2.5% (by weight) tobacco-derived United States Pharmacopeia (USP) Grade nicotine, 306.0 ± 9.2 mg/mL PG (mean ± SD), 600.0 ± 21.3 mg/mL glycerol (mean ± SD), flavor ingredients (non-menthol, proprietary formulation) and water. Group II study participants used a prototype cartridge-based EVP (supplied by Altria Client Services LLC, Richmond, VA, USA). The e-liquid in the cartridges consisted of 2.4% (by weight) tobacco-derived USP Grade nicotine, 465.0 ± 4.5 mg/mL PG (mean ± SD), 480.0 ± 6.7 mg/mL glycerol (mean ± SD), flavor ingredients (non-menthol, proprietary formulation) and water. Group III study participants used the Compact eGo RBC tank device (900 mAh, 3.7 volt battery, manufacturer Shenzhen IVPS Co, Ltd., Shenzhen, China) with their own preferred e-liquid with nicotine (concentrations ranging from 5.92 to 23.9 mg/mL), PG (concentrations ranging from 362 to 688 mg/mL) and glycerol (concentrations ranging from 212 to 700 mg/mL). Group IV study participants used their own brand of non-menthol CC.

### 2.2. Analytical Procedures

Nicotine in both the XAD-7 and solid surface samples was analyzed with an Agilent Model 7890 Gas Chromatograph (GC) coupled to an Agilent Model 5975C Mass Selective Detector (Agilent Technologies, Santa Clara, CA, USA). The XAD-7 and solid surface extracts were analyzed following ISO 16200-1 [48]. PG and glycerol in both the XAD-7 and solid surface samples were analyzed with an Agilent Model 6890 GC equipped with a Flame Ionization Detector. The XAD-7 and solid surface extracts were analyzed following ISO 16200-1 [48]. VOCs were analyzed with an Agilent Technologies Model 6890, GC equipped with a 5973 Mass Selective Detector (ISO 16000-6) [49]. Carbonyls were analyzed with an Agilent Model 1100 High Performance Liquid Chromatograph with a DAD-UV detector operated at 365 nm (ISO-16000-3) [50]. Selected trace elements were determined by Inductively Coupled Plasma-Mass Spectrometry with a Perkin Elmer DRC-e ICP-MS (EPA IO-2.1 and 3.5) [51,52]. All results for RAS are given as μg/m^3^. The limit of quantification (LOQ) for each compound is shown in Table 1. Results for surface samples are given as μg/cm^2^. 

### 2.3. Statistical Analyses

This study was designed to compare the level of each chemical between baseline and product use conditions, for each product. The analysis included all 37 subjects who were enrolled in the study and provided exposure data for at least one period. Descriptive statistics were provided for absolute change from baseline in RAS constituents by study group and product use condition (pre-specified use and ad libitum use). Paired t-tests were used to compare the mean level of each compound in room air and in surface samples at baseline and during product use, for all groups. Values found below the LOQ (BLOQ) were set to zero. The values were not calculated for instances where both the baseline/background values as well as the room air levels during EVP use were <LOQ and reported as not calculated (N.C.). Significance was set at *p* < 0.05. All analyses were performed with SAS 9.2 (SAS Institute, Cary, NC, USA).

## 3. Results

### 3.1. Study Participants Disposition

A total of 37 study participants (23 males and 14 females) were enrolled. The mean age was 39.5 years and ranged from 21 to 63 years. The BMI was similar in all groups and ranged from 19 to 39 kg/m^2^. 

Group I participants consisted of four exclusive e-vapor (two cartridge-based and two tank-based) users, five concurrent e-vapor (four tank) users and CC smokers. The average daily usage for cartridge, tank e-liquid and conventional cigarettes were 1.17 cartridge/day, 3.25 mL/day and 5.4 CPD, respectively.

Group II participants consisted of six exclusive e-vapor (one cartridge-based and five tank-based) users, three concurrent e-vapor (tank) users and CC smokers. The average daily usage for cartridge, tank e-liquid and CC were 1.33 cartridge/day, 3.43 mL/day and 3.0 CPD, respectively.

Group III participants consisted of seven exclusive e-vapor (tank) users and two concurrent e-vapor (tank) users and CC smokers. The average daily usage for tank e-liquid and CCs were 4.13 mL/day and 2.0 CPD, respectively.

Group IV participants consisted of nine exclusive CC smokers and one concurrent cartridge-based e-vapor user and CC smoker. The average daily CC consumption was 15.5 CPD, and the daily usage for cartridge was 0.14 cartridge/day for the subject who used e-vapor.

No clinically relevant changes in vital signs (blood pressure, heart rate and respiratory rate) after product use were observed for any study participant. Two study participants reported a total of two mild adverse events (AEs) during the study. None of the AEs were serious (as determined by the PI) or resulted in withdrawal from the study. One study participant in Group II experienced dyspepsia after using the product and one study participant in Group III experienced headache one day prior to product use. Both events resolved on the same day as onset. In the opinion of the PI, dyspepsia was considered to be possibly related to the product.

One study participant in Group I withdrew from the study approximately two hours after the start of the ad libitum session due to a family emergency. All remaining 36 study participants completed the study according to protocol.

### 3.2. Product Use

The total number of puffs taken were 720 (pre-specified use) and 1224 and 747 (ad libitum use) for Groups I and II respectively and 1649 from Group III. Study participants in Group I consumed a total of 2472 mg of e-liquid during the four hours of product use under pre-specified conditions, and a total of 4211 mg during the ad libitum session. Group II study participants consumed a total of 3324 mg during pre-specified use and 2237 mg during ad libitum use. Group III study participants consumed a total of 12,523 mg of e-liquid during ad libitum use. Group IV study participants smoked a total of 45 cigarettes during the ad libitum session.

### 3.3. Levels of Selected Constituents in RAS

Of the 34 constituents measured in room air, 23 (nicotine, glycerol, arsenic, cadmium, crotonaldehyde, *o*-tolualdehyde, butyraldehyde, *m*- and *p*-tolualdehyde, benzaldehyde, propionaldehyde, isovaleraldehyde, hexaldehyde, valeraldehyde, 2,5-dimethylbenzaldehyde, acrolein, 1,3-butadiene, furan, ethylene oxide, vinyl chloride, propylene oxide, nitromethane, 2-nitro-propane and vinyl acetate) were BLOQ at baseline. Out of these, 11 (arsenic, cadmium, *o*-tolualdehyde, *m*- and *p*-tolualdehyde, propionaldehyde, acrolein, ethylene oxide, propylene oxide, nitromethane, 2-nitropropane and vinyl acetate) remained BLOQ during product use for all groups. All other constituents could be quantified in RAS at least during one condition. When EVPs were used, 13 out of 34 constituents were measurable above the LOQ. When CCs were used, 21 out of 34 constituents were measured above the LOQ. The mean level of each compound during each test condition is shown in supplementary material (Table S1), as well as the individual raw data for each sampling port (Table S2). Table 1 shows the mean changes from baseline levels to product use conditions, for each compound and each product. The values were corrected for background levels only for formaldehyde, acetaldehyde, acetone and methyl ethyl ketone where background levels were above the LOQ. These additional steps were taken to measure the background air levels for carbonyls for each study group to ensure that we appropriately measure potential confounding sources for these chemicals. 

#### 3.3.1. Nicotine, PG and Glycerol

**Figure 3:**
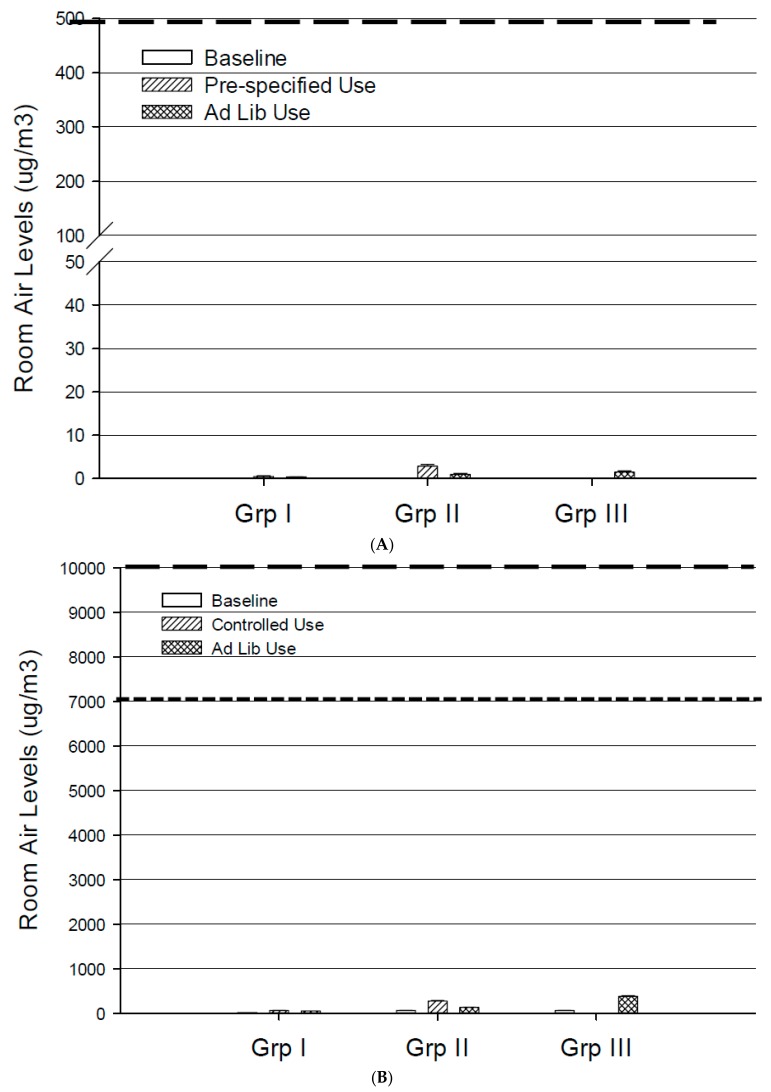

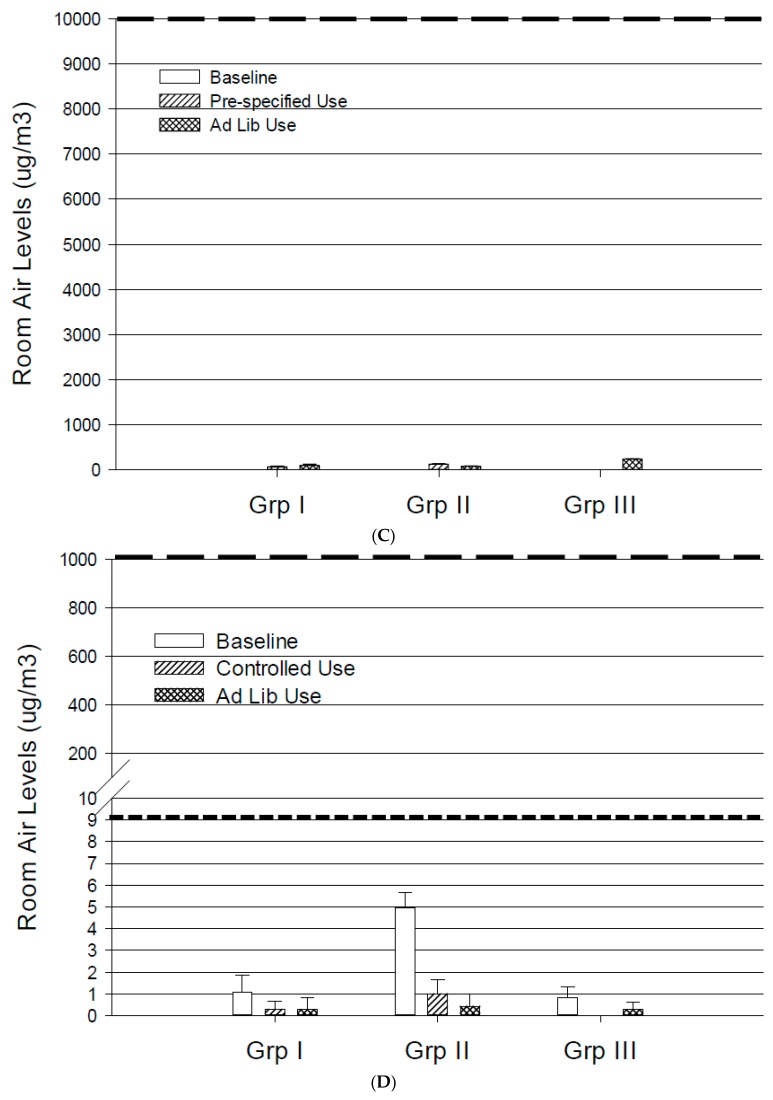
Mean levels of nicotine (**A**); propylene glycol (**B**); glycerol (**C**) and formaldehyde (**D**) in RAS at baseline and from the use of the two EVPs (Groups I and II) and the tank product (Group III) over four hours of use. The dashed lines represent limits for air contaminants set by various agencies (value shown as 8-h time weighted average permissible exposure limit); nicotine: Occupational Safety and Health Administration (OSHA) limit of 500 μg/m^3^; PG: American Industrial Hygiene Association (AIHA) limit of 10,000 μg/m^3^ (wide dashed lines) and Office of Environmental Health Hazard Assessment, California Environmental Protection Agency (OEHHA) limit of 7000 μg/m^3^ (narrow dashed lines); glycerol: OSHA limit of 10,000 μg/m^3^; and formaldehyde: OSHA limit of 980 μg/m^3^ (wide dashed lines) and OEHHA limit of 9 μg/m^3^ (narrow dashed lines).

At baseline, measurable levels of PG (above the LOQ) were detected in the room. Levels of nicotine, PG and glycerol were statistically significantly higher than baseline during all product use conditions for all products, except glycerol that remained BLOQ during CC use. Of all products, the tank device produced the highest difference from baseline in the level of PG and glycerol, with mean changes from baseline of 317.06 and 242.00 μg/m^3^, respectively (Table 1). The highest level of nicotine in RAS was found for Group IV, where it reached a mean level of 40.65 μg/m^3^. The mean nicotine levels ranged from 0.38 to 2.83 μg/m^3^ (Table S1 and Figure 3A) during EVP use. Figure 3B,C show the mean PG and glycerol levels for the three groups under different use conditions.

#### 3.3.2. Carbonyls

Measurable levels (above the LOQ) were observed for formaldehyde, acetaldehyde, acetone and methyl ethyl ketone in the room air at baseline. Nine out of the 15 measured carbonyls were statistically significantly higher than baseline when CCs (Group IV) were used in the EC; acetaldehyde, formaldehyde and acetone showed the highest differences from baseline with mean changes of 105.16, 49.74 and 45.70 μg/m^3^, respectively. For Group I, only hexaldehyde was statistically significantly higher than baseline, when the EVP was used ad libitum. For Group II, none of the carbonyls were significantly higher during product use compared with baseline. Four carbonyls were statistically significantly lower than at baseline. For Group III, only acetaldehyde was significantly higher than at baseline (Table 1). Figure 3D shows the level of formaldehyde in RAS at baseline and for Groups I–III under different use conditions.

#### 3.3.3. Volatile Organic Constituents

Detectable levels of benzene, isoprene, toluene and ethylbenzene were observed at baseline. Statistically significant changes from baseline were observed in six of the 12 VOCs for Group IV. Isoprene showed the highest change, with a difference of 167.81 μg/m^3^ between baseline and CC use. When EVPs were used, statistically significant changes from baseline were observed for benzene, isoprene and toluene. Some observations were higher than baseline, whereas other levels were significantly lower than baseline. The largest differences were observed for toluene both under the pre-specified use conditions; the change from baseline was +1.90 μg/m^3^ for Group I, whereas for Group II was −2.02 μg/m^3^ (Table 1).

#### 3.3.4. Selected Trace Elements

Chromium was significantly lower than baseline during both product use conditions for Group II. No significant changes between baseline and product use were observed for nickel. Levels of cadmium and arsenic were not quantifiable in the room air under any of the product use conditions (Table 1).

### 3.4. Nicotine, PG and Glycerol in Surface Samples

At baseline, the level of nicotine, PG and glycerol in surface samples was BLOQ for all groups. Table S3 presents the levels of nicotine, PG and glycerol measured for each surface sample during each product use condition. Table 2 shows the mean changes in the level of nicotine, PG and glycerol on surface samples, from baseline to each product use condition. 

The levels of nicotine, although measurable in 2/24 samples (four samples for each of the six use conditions), were not statistically significantly different compared to baseline. PG was measurable in 6/24 samples when EVPs were used, but the mean levels were not statistically significantly different between baseline and product use conditions. PG was not measurable in surface samples when CCs were used. Glycerol was measurable in 11/24 samples, when EVPs were used. When Group III study participants used the tank product, the mean level of glycerol on surface samples, measured at 0.35 μg/cm^2^, was statistically significantly higher than at baseline. Glycerol was not detected when CCs were used.

## 4. Discussion

The use of the study EVPs, cartridge- and tank-based, did not generate chemicals at levels that could likely pose health concerns for non-users under the study conditions. Given that nicotine, PG and glycerol are the major constituents in the product, it is not surprising that measurable levels are found in a room where EVPs are used. However the cumulative measurement of levels of these constituents over four hours in the room were relatively small and several-fold below the current published limits for workplace exposure to airborne contaminants [53,54,55]. Room air formaldehyde levels from EVPs were not detectable above the background and/or baseline levels. These results corroborate our analytical chemistry study of aerosol generated from the MarkTen^®^ prototype e-cigarette [7] and previous report from two studies where indoor vaping of MarkTen^®^ prototype e-cigarette did not produce chemicals above quantifiable levels or different from background levels using standard industrial hygiene collection techniques and analytical methods [31].

### 4.1. Room Air Levels from Use of Different Tobacco Products

In Group I, during MarkTen^®^ use, the mean differences between baseline and product use were statistically significant under pre-specified and/or ad libitum conditions for 7 out of 34 chemicals (nicotine, PG, glycerol, hexaldehyde, benzene, isoprene and toluene). The baseline room air levels of benzene, without any product use, were highly variable, the average levels ranging from 0.5843 μg/m^3^ (Group I) to 2.2685 μg/m^3^ (Group IV) (see Supplementary Table S1). During EVP use, the room air levels of benzene were higher than baseline only for Group 1, however the differences were small and in previous studies this chemical was not detected in aerosol from similar devices [7,10]. Furthermore, the average room air levels of benzene were higher under pre-specified use conditions (0.8338 μg/m^3^) than under ad lib use (0.540 μg/m^3^) compared to the baseline levels of 0.5483 μg/m^3^ (Table S1). These values are not bioplausible given that the number of puffs taken during the pre-specified (*n* = 720) were far fewer than under ad lib use conditions (*n* = 1224). Therefore the apparent higher room air levels of benzene from half as many puffs is likely due to inherent background variability from other sources. Similarly only four out of 34 constituents (nicotine, PG, glycerol and isoprene, and only under pre-specified use conditions) were statistically significantly higher than baseline with the cartridge-based prototype EVP use in Group II. The levels of isoprene during EVP use were within the range of baseline levels across the groups from 6.4588 to 7.8865 μg/m^3^. No detectable isoprene has been detected in machine generated aerosols from similar products [7], suggesting that the isoprene levels observed during EVP use could potentially be an artifact. 

The room air levels for five out of 34 constituents in Group II were lower under EVP use conditions compared to baseline, confirming the inherent variability in these measurements. The higher baseline levels explain the apparent negative values for the mean change from baseline for benzene and toluene during the EVP use. These observations suggests that while a sensitive analytical method may be able to detect very low levels of constituents, the room air levels should be interpreted with caution due to the changes in background levels of the target analytes.

In Group III, the mean change from baseline for room air levels, from tank-based EVP use, were statistically significant for 6 out of 34 constituents (nicotine, PG, glycerol, acetaldehyde, benzene and isoprene). The average value for one of the constituents in Group III, benzene, during ad libitum product use was higher than baseline.

On the other hand for CC use in Group IV, 17 out of 34 constituents were statistically significantly higher, confirming the sensitivity of the sample collection and analytical methodology.

### 4.2. Levels of Different Groups of Chemicals

The cumulative four hour room air levels of the chemicals measured above the LOQ were relatively small. For example, the levels of formaldehyde were highly variable and during EVP use were lower than the background or baseline levels. Although comparison to occupational exposure values has been challenged [56], a simple comparison provides a perspective. The cumulative four hour room air levels of formaldehyde measured during different EVP product use were several-fold below the maximum occupational exposure limit of 370 μg/m^3^ set by Federal Republic of Germany (DFG) [57] and the ACGIH TLV of 120 μg/m^3^ as well as the limit of 980 μg/m^3^ set by the Occupational Safety and Health Administration (OSHA) [54]. Occupational exposure values represent the upper limit values that are not expected to adversely affect workers’ health over their working lives (8 h per day, 5 days per week) and do not specifically include any susceptible sub-groups or populations [56]. It should also be recognized that various guidelines exist for chemical constituents in indoor air including levels of formaldehyde [58,59]. The recommended indoor levels of formaldehyde range from 100 μg/m^3^ [59] to 9 μg/m^3^ [58] depending on the length of exposure and there has been considerable debate regarding the appropriateness of these levels [60,61]. For example, Salthammer et al. [60] concluded that “Moreover, it seems questionable whether formaldehyde concentrations lower than 20 μg/m^3^ can be permanently achieved under normal living conditions in urban and rural environments.” Our study demonstrated that the EVP products tested resulted in no increase in background levels of formaldehyde compared to an order of magnitude increase in formaldehyde levels when CC were used. Even if we used the most conservative estimate of OEHHA from the California Environmental Protection Agency of 9 μg/m^3^ the comparisons should be made in the context that the formaldehyde room air levels from EVP use were all below background.

We performed the study in a specialized chamber that has been previously validated and designed as a stand-alone unit for controlled air exchange rates. We also had established several sampling stations within the room to obtain an accurate assessment of various chemicals in the room air after use of EVPs and CCs. Closer review of the individual sampling stations (see Supplementary Table S2) indicated that there were no patterns for differences in room air levels in different locations in the room. The sampling ports within the breathing sphere of the participants were not directionally different from the sampling ports farthest in the EC. These observations suggest that other than the three major constituents of EVPs (nicotine, PG and glycerol), minimal if any levels of other chemicals should be expected in a room where EVPs are used.

We used experienced EVP users, rather than smoking machines to generate secondhand aerosol, to best represent real-life conditions. We also measured all chemicals in the chamber RAS with (baseline) and without (background) study participants present in the room, in order to account for other potential human and non-human sources of room air toxicants. 

Nicotine was not detected in room air at baseline, even when smokers of CCs were present in the EC. However, when the tested EVPs were used, the level of nicotine in room air was quantified at 0.38 to 2.83 μg/m^3^, and was significantly higher than at baseline for each test condition. This is in broad agreement with findings from Czogala et al. [13] and Schober et al. [35], who found nicotine levels ranging from 0.6 to 6.23 μg/m^3^ when study participants used different nicotine-containing EVPs. This is also consistent with the work of St. Helen et al. [18], who found that EVP users exhale, on average, only 6% of the inhaled nicotine. As can be seen in Figure 3A, a simple comparison of the 4-h cumulative observed in our study provides some context when compared to the 8-h time weighted average (TWA) exposure limits set by OSHA. The assumptions e.g., cumulative value vs. 8-h TWA and purpose of the OSHA limits should be borne in mind when making the comparisons. The relatively low levels of nicotine observed in the room despite consumption of an estimated 54 to 221 mg of nicotine (based on the amount of e-liquid consumed) suggests that a significant fraction of the nicotine is absorbed by users and very little is exhaled out in the environment. The use of CCs produced much higher levels of nicotine in room air than EVPs, with mean nicotine levels of 40.65 μg/m^3^ in our study that were comparable to that observed by Schober et al.’s study (31.6 μg/m^3^) [35]. Our study presents data collected during pre-specified and ad libitum use of various types of EVPs and cigarettes reflecting a spectrum of use conditions. These observations may be considered more relevant than machine-generated aerosols which significantly overestimates the room levels of nicotine and should be interpreted with extreme caution. 

Regarding carrier constituents, both PG and glycerol were also significantly higher than at baseline when all EVPs were used. Significant levels of PG were detected in the room during the baseline period, without product use, demonstrating the ubiquitous nature of PG [62,63]. Higher levels of PG and glycerol (317 and 242 μg/m^3^ respectively) were observed during use of tank-based EVPs. These levels were generally similar in range as reported in another study when study participants used a tank device ad libitum (PG ~ 395 μg/m^3^ and glycerol ~ 81 μg/m^3^) [35]. However, PG was BLOQ in another study where a study participant used a tank device in a test chamber [16]. These differences could be due to differences in delivery of the tanks used in each study, the differing PG:glycerol ratio in the tank devices, or the sensitivity of the analytical methodologies used. A simple comparison of our four hour cumulative measurements of PG and glycerol with the AIHA Workplace Environmental Exposure Level for PG and OEHHA 2016 indoor air guidelines (Figure 3B) and OSHA Permissible Exposure Limit for glycerol (Figure 3C) demonstrate that our four hour cumulative measurements of PG and glycerol are orders of magnitude lower [53,54]. In our study, the use of CCs did not produce detectable levels of glycerol, but produced levels of PG that were in the range of the EVP. The CCs tested in the study were commercial products and levels of glycerol and PG were not measured. Acrolein has been reported as a thermal degradation by-product of glycerol [64]. The lack of detectable acrolein suggests that even if glycerol were pyrolyzed, insufficient amount is exhaled in the air to be of any potential consequence.

Generally greater chemicals levels were measured in room air after use of the tank devices than the cartridge-based products. The greater measured levels may be due to the ~3–5-fold higher amount of e-liquid consumed by the tank users. The change from baseline was negative for some of the chemicals (e.g., formaldehyde and acetaldehyde) suggesting that the room air levels were higher at baseline and/or background. These observations are consistent with other reports of significant background levels present from other sources, including our own study [31]. For example, formaldehyde exposure can occur from environmental sources (combustion processes, building materials, and tobacco smoke) or in occupational settings (furniture, textile, and construction industries) [65]. Formaldehyde is also formed endogenously in the cellular metabolic pathways [66] and has been detected in exhaled breath air samples [67,68]. Our results clearly demonstrate that the levels of formaldehyde produced under regular EVP use are below the normal background levels. Under the conditions of the study and with the types of EVPs used, little if any toxicity may be anticipated from exposure to the exhaled chemicals measured. 

It is worth noting that none of the four heavy metals were detected significantly above the baseline levels during EVP use. These observations suggest that metals from some of EVP design components, as reviewed in a recent publication [69], are not released in the room, at least for the types of products tested in this study. Out of all the other tested constituents, acetaldehyde, acetone, hexaldehyde, methyl ethyl ketone, benzene, isoprene and toluene could be quantified at some instances when EVPs were used. The levels of these constituents were either above or below the baseline levels, suggesting intrinsic variability either from analytical measurements or background environmental variability. The latter is not uncommon given that VOCs and aldehydes have often been detected in commercial and residential buildings [70,71,72]. 

### 4.3. Potential Limitations

The results of this study should be interpreted relative to some of the limitations. First, given that EVPs are rapidly evolving and the most recent generation such as “direct drip atomizers” [73] may have differential aerosol characteristics and room air level results from the use of such products may need to be investigated. However, the results from this study are at least applicable to the cartridge-based and tank-based categories of EVPs tested. 

Second, the study was conducted under a single condition of room size and air handling, and does not provide information for different conditions e.g., potential exposure in a closed car. The air exchange rate used in this study was based on typical conditions for office buildings and the data should be interpreted with caution since the room air levels of the chemicals could potentially be higher under a scenario of lower exchange rates of a residential environment. We believe that rather than conducting multiple studies under different conditions, the observations from this study will provide parameters for a computational model [74] that should readily allow changes of the exposure conditions (multiple people present in the room, different size room, and different air exchange rates etc.). 

Third, the relatively small magnitude of the reported values for some chemicals should be interpreted with caution due to inherent variability in the background/baseline values, particularly airborne chemicals due to multiple other confounding sources. The design of this study of including background and baseline measurements may help minimize the possibility of reporting artifactual data for these select chemicals. 

Finally, some researchers report measurable surface levels of nicotine from tank-based EVPs when machine-generated aerosol was released in the air [40]. Observations from machine-generated aerosols are a gross overestimation since nicotine uptake by the user is not taken into consideration. We report that surface nicotine levels in our study during EVP use were not statistically significantly higher compared to baseline. However these results should be interpreted with caution since the limited duration of product use may not reflect the potential accumulation over time. Additionally the petri-dish collection method may have limited utility since actual indoor environments contain many more types of surfaces and materials, which are much more porous and may serve as a reservoir for semi-volatile compounds. However, the measurement of the residue deposited on the petri-dish does provide information regarding likelihood of thirdhand exposure as it reflects the cumulative amount deposited over time. Perhaps the report by Bush et al. might be more realistic since the authors measured surface wipe samples from homes of EVP users [39]. They report that nicotine levels were not significantly different in homes of EVP users compared to homes of non-tobacco users.

## 5. Conclusions

Overall, these findings indicate that under the study conditions, with the products tested, the room air levels of the select measurable chemicals during the use of EVPs were relatively small and were several-fold below the current occupational regulatory and consensus limits. The study results could provide further information for the ongoing debate regarding the potential health impact for exposing non-users to chemicals from EVP use.

## Figures and Tables

**Table 1:** Mean changes in the level of nicotine, PG, glycerol and selected constituents in RAS from baseline to product use conditions (μg/m^3^).

RAS Constituent	LOQ * (μg/m^3^)	Group I (*n* = 9)		Group II (*n* = 9)		Group III (*n* = 9)	Group IV (*n* = 10)
		Pre-Specified	Ad libitum	Pre-Specified	Ad libitum	Ad libitum	Ad libitum
Nicotine	0.25	**0.48 ± 0.16**	**0.38 ± 0.07**	**2.83 ± 0.44**	**0.96 ± 0.22**	**1.47 ± 0.32**	**40.65 ± 6.40**
Propylene glycol	3.63	**44.86 ± 3.84**	**33.06 ± 1.97**	**211.51 ± 14.23**	**68.51 ± 4.58**	**317.06 ± 12.45**	**56.21 ± 4.22**
Glycerol	4.11	**67.89 ± 16.81**	**98.90 ± 28.14**	**126.75 ± 12.71**	**78.65 ± 8.75**	**242.00 ± 7.62**	NC
Arsenic	0.12	n/a	n/a	n/a	n/a	n/a	n/a
Cadmium	0.12	n/a	n/a	n/a	n/a	n/a	n/a
Chromium	0.12	0.01 ± 0.15	0.012 ± 0.11	**−0.14 ± 0.01**	**−0.14 ± 0.01**	−0.05 ± 0.08	0.04 ± 0.06
Nickel	0.12	0.07 ± 0.08	−0.04 ± 0.07	−0.17 ± 0.13	−0.20 ± 0.14	NC	NC
Formaldehyde ^‡^	0.62	−0.83 ± 0.69	−0.78 ± 0.64	**−3.96 ± 0.45**	**−4.53 ± 0.45**	−0.55 ± 0.82	**49.74 ± 4.18**
Crotonaldehyde	0.62	NC	NC	NC	NC	NC	1.09 ± 0.78
*o*-Tolualdehyde	0.62	n/a	n/a	n/a	n/a	n/a	n/a
Acetaldehyde ^‡^	0.62	−0.36 ± 0.57	−0.54 ± 0.47	**−1.98 ± 0.87**	0.12 ± 3.94	**1.10 ± 0.51**	**105.16 ± 5.24**
Butyraldehyde	0.70	NC	NC	NC	NC	NC	**6.12 ± 0.86**
*m*- and *p*-Tolualdehyde	1.24	n/a	n/a	n/a	n/a	n/a	n/a
Acetone ^‡^	0.62	−3.72 ± 9.27	1.91 ± 12.22	−0.96 ± 8.09	**−8.45 ± 3.54**	n/a	**45.70 ± 7.03**
Benzaldehyde	0.62	NC	NC	NC	NC	NC	0.53 ± 1.06
Propionaldehyde	0.80	n/a	n/a	n/a	n/a	n/a	n/a
Isovaleraldehyde	0.62	NC	NC	NC	NC	NC	**2.10 ± 0.57**
Hexaldehyde	1.20	NC	**2.07 ± 0.52**	NC	NC	NC	**3.27 ± 0.37**
Valeraldehyde	0.62	NC	NC	NC	NC	NC	**6.10 ± 0.89**
2,5-Dimethylbenzaldehyde	0.88	NC	NC	NC	NC	NC	**4.54 ± 0.84**
Methyl ethyl ketone ^‡^	1.45	0.69 ± 1.20	0.02 ± 0.05	−1.71 ± 1.69	−0.88 ± 2.34	0.10 ± 0.20	**16.97 ± 1.84**
Acrolein	0.62	n/a	n/a	n/a	n/a	n/a	n/a
1,3-Butadiene	0.19	NC	NC	NC	NC	NC	10.56 ± 0.57
Benzene	0.28	**0.25 ± 0.02**	**0.04 ± 0.03**	**−0.75 ± 0.18**	**−0.73 ± 0.14**	**0.15 ± 0.05**	**13.24 ± 0.46**
Isoprene	0.24	**1.72 ± 0.31**	−0.41 ± 1.70	**0.55 ± 0.15**	−0.18 ± 1.77	**1.84 ± 0.17**	**167.81 ± 7.81**
Toluene	0.33	**1.90 ± 0.38**	**0.43 ± 0.23**	**−2.02 ± 0.25**	**−1.53 ± 0.31**	0.13 ± 0.46	**30.83 ± 1.20**
Furan	0.18	NC	NC	NC	NC	NC	**12.08 ± 0.50**
Ethylene oxide	0.63	n/a	n/a	n/a	n/a	n/a	n/a
Vinyl chloride	0.22	NC	NC	NC	NC	NC	0.11 ± 0.23
Propylene oxide	0.83	n/a	n/a	n/a	n/a	n/a	n/a
Nitromethane	4.33	n/a	n/a	n/a	n/a	n/a	n/a
2-Nitropropane	6.33	n/a	n/a	n/a	n/a	n/a	n/a
Vinyl acetate	6.11	n/a	n/a	n/a	n/a	n/a	n/a
Ethylbenzene	0.38	NC	NC	NC	NC	NC	**3.52 ± 0.15**

PG: Propylene glycol; RAS: Room air sample; LOQ: Limit of quantification; *: LOQ is based on the actual air volume sampled; NC: not calculated (values < LOQ). Note: Values are means ± standard deviations of four air samplers placed in the EC. *n* is the number of study participants present in the EC for each group. Statistically significant (*p*-value < 0.05) differences between baseline and product use condition are highlighted in bold font. ^‡^ Values were corrected for background levels. n/a: not applicable, values for the constituent were below the LOQ for all conditions, or compound was not quantifiable due to unknown matrix interferences.

**Table 2:** Mean changes in the level of nicotine, PG and glycerol in surface samples from baseline to product use conditions (μg/cm^2^).

Condition	LOQ (μg/cm^2^)	Group I	Group II	Group III	Group IV
		(*n* = 9)	(*n* = 9)	(*n* = 9)	(*n* = 10)
**Nicotine**	0.001				
Pre-specified		0.001 ± 0.002	NC	n/a	n/a
Ad libitum		NC	NC	NC	0.001 ± 0.002
**Propylene glycol**	0.019				
Pre-specified		0.02 ± 0.01	0.005 ± 0.010	n/a	n/a
Ad libitum		NC	0.01 ± 0.01	NC	NC
**Glycerol**	0.02				
Pre-specified		NC	0.07 ± 0.02	n/a	n/a
Ad libitum		0.02 ± 0.01	NC	**0.35 ± 0.12**	0.00 ± 0.00

Note: Values are means ± standard deviations of four surface samplers placed in the EC. *n* is the number of study participants present in the EC for each group. NC: not calculated (values < LOQ). Statistically significant (*p*-value < 0.05) differences between baseline and product use condition are highlighted in bold font. n/a: not applicable.

## Supplementary Materials

The following are available online at www.mdpi.com/1660-4601/14/9/969/s1, Table S1: Absolute levels of RAS constituents (μg/m^3^) measured during background (where detected), baseline and product use, Table S2: Absolute levels of RAS constituents (μg/m^3^) measured during background (where detected), baseline and product use conditions, for all groups, for each of the four sampling port, Table S3: Absolute levels of nicotine, propylene glycol and glycerol (μg/cm^2^) in each surface sample location during each product use condition.

## Abbreviations

AE	adverse event
BLOQ	below the limit of quantification
BMI	body mass index
CC	conventional cigarette
CPD	cigarettes per day
EC	exposure chamber
EVP	electronic vapor product
GC	gas chromatograph
HPHC	harmful and potentially harmful constituent
LOQ	limit of quantification
PG	propylene glycol
RAS	room air sample
VOC	volatile organic compound
