# Supplementary material for: Determination of Selected Chemical Levels in Room Air and on Surfaces after the Use of Cartridge- and Tank-Based E-Vapor Products or Conventional Cigarettes
Source: Int J Environ Res Public Health. 2017 Aug 28;14(9):969. doi: 10.3390/ijerph14090969 (PMC5615506; doi:10.3390/ijerph14090969)
Supplement: Supplementary file 1 [file ijerph-14-00969-s001.pdf]

Table S1. Absolute levels of RAS constituents (μg/m^3) measured during background (where detected), baseline and product use.

**Constituent** (LOQ μg/m^3) Condition	**Group I** (N=9)	**Group II** (N=9)	**Group III** (N=9)	**Group IV** (N=10)
**Nicotine** (0.248)				
Baseline	<LOQ	<LOQ	<LOQ	<LOQ
Pre-defined	0.4763 ± 0.1618	2.8275 ± 0.4371	n/a	n/a
Ad libitum	0.3810 ± 0.0694	0.9595 ± 0.2232	1.4725 ± 0.3177	40.6500 ± 6.3966
**Propylene glycol** (3.63)				
Baseline	17.338 ± 2.199	63.238 ± 4.015	61.938 ± 6.216	9.763 ± 1.225
Pre-defined	62.200 ± 5.144	274.750 ± 12.230	n/a	n/a
Ad libitum	50.400 ± 3.186	131.750 ± 3.862	379.000 ± 15.556	65.975 ± 5.161
**Glycerol** (4.11)				
Baseline	<LOQ	<LOQ	<LOQ	<LOQ
Pre-defined	67.888 ± 16.806	126.750 ± 12.712	n/a	n/a
Ad libitum	98.900 ± 28.142	78.650 ± 8.745	242.000 ± 7.616	<LOQ
**Arsenic** (0.123)				
Baseline	<LOQ	<LOQ	<LOQ	<LOQ
Pre-defined	<LOQ	<LOQ	n/a	n/a
Ad libitum	<LOQ	<LOQ	<LOQ	<LOQ
**Cadmium** (0.123)				
Baseline	<LOQ	<LOQ	<LOQ	<LOQ
Pre-defined	<LOQ	<LOQ	n/a	n/a
Ad libitum	<LOQ	<LOQ	<LOQ	<LOQ
**Chromium** (0.123)				
Baseline	0.0613 ± 0.0707	0.1370 ± 0.0059	0.1203 ± 0.0825	0.0655 ± 0.0757
Pre-defined	0.0665 ± 0.0769	<LOQ	n/a	n/a
Ad libitum	0.0728 ± 0.0840	<LOQ	0.0688 ± 0.0799	0.1040 ± 0.0696
**Nickel** (0.123)				
Baseline	0.0358 ± 0.0715	0.2048 ± 0.1378	<LOQ	<LOQ
Pre-defined	0.1100 ± 0.0746	0.0313 ± 0.0625	n/a	n/a
Ad libitum	<LOQ	<LOQ	<LOQ	<LOQ
**Formaldehyde** (0.619)				
Background before baseline	2.740 ± 0.890	0.725 ± 1.450	2.603 ± 0.350	2.510 ± 0.440
Baseline	3.690 ± 0.289	5.685 ± 1.710	3.433 ± 0.204	4.375 ± 0.108
Background before pre-defined	6.043 ± 1.922	3.970 ± 0.721	n/a	n/a
Pre-defined	5.355 ± 0.078	4.973 ± 0.340	n/a	n/a
Background before *ad libitum*	11.795 ± 2.661	6.238 ± 1.479	3.728 ± 0.296	3.500 ± 0.087
Ad libitum	9.160 ± 0.995	5.823 ± 0.123	3.878 ± 0.509	55.100 ± 4.113
**Crotonaldehyde** (0.619)				
Baseline	<LOQ	<LOQ	<LOQ	<LOQ

Pre-defined	<LOQ	<LOQ	n/a	n/a
Ad libitum	<LOQ	<LOQ	<LOQ	1.0935 ± 0.7839
**o-Tolualdehyde** (0.0149)				
Baseline	<LOQ	<LOQ	<LOQ	<LOQ
Pre-defined	<LOQ	<LOQ	n/a	n/a
Ad libitum	<LOQ	<LOQ	<LOQ	<LOQ
**Acetaldehyde** (0.619)				
Background before baseline	3.0125 ± 0.7023	0.6450 ± 1.2900	4.8650 ± 0.4876	4.6625 ± 0.1702
Baseline	3.4638 ± 0.2354	2.7975 ± 0.4545	3.8275 ± 0.1909	4.1025 ± 0.1748
Background before pre-defined	5.2800 ± 1.9407	3.6725 ± 0.5538	n/a	n/a
Pre-defined	3.9313 ± 0.3089	3.6775 ± 0.3652	n/a	n/a
Background before *ad libitum*	8.7350 ± 1.2678	5.4750 ± 0.6368	4.2850 ± 0.3608	5.3400 ± 0.5995
Ad libitum	6.4075 ± 0.6394	7.7288 ± 4.4290	5.3825 ± 0.4129	110.5000 ± 5.3229
**Butyraldehyde** (0.698)				
Baseline	<LOQ	<LOQ	<LOQ	<LOQ
Pre-defined	<LOQ	<LOQ	n/a	n/a
Ad libitum	<LOQ	<LOQ	<LOQ	6.118 ± 0.860
**m- and p-Tolualdehyde** (1.240)				
Baseline	<LOQ	<LOQ	<LOQ	<LOQ
Pre-defined	<LOQ	<LOQ	n/a	n/a
Ad libitum	<LOQ	<LOQ	<LOQ	<LOQ
**Acetone** (0.619)				
Background before baseline	21.618 ± 29.558	3.938 ± 7.875	NR	27.050 ± 8.662
Baseline	16.013 ± 0.802	21.000 ± 5.314	NR	40.925 ± 4.148
Background before pre-defined	35.500 ± 21.324	14.943 ± 5.394	n/a	n/a
Pre-defined	25.863 ± 0.477	31.050 ± 4.118	n/a	n/a
Background before *ad libitum*	41.350 ± 17.666	21.625 ± 10.341	NR	19.025 ± 5.405
Ad libitum	45.588 ± 3.171	30.238 ± 5.025	NR	78.600 ± 6.044
**Benzaldehyde** (0.619)				
Baseline	<LOQ	<LOQ	<LOQ	<LOQ
Pre-defined	<LOQ	<LOQ	n/a	n/a
Ad libitum	<LOQ	<LOQ	<LOQ	0.5300 ± 1.0600
**Propionaldehyde** (0.798)				
Baseline	<LOQ	<LOQ	<LOQ	<LOQ
Pre-defined	<LOQ	<LOQ	n/a	n/a
Ad libitum	NR	<LOQ	<LOQ	<LOQ
**Isovaleraldehyde** (0.619)				
Baseline	<LOQ	<LOQ	<LOQ	<LOQ
Pre-defined	<LOQ	<LOQ	n/a	n/a
Ad libitum	<LOQ	<LOQ	<LOQ	2.095 ± 0.567
**Hexaldehyde** (1.200)				
Baseline	<LOQ	<LOQ	<LOQ	<LOQ
Pre-defined	<LOQ	<LOQ	n/a	n/a

Ad libitum	2.0698 ± 0.5217	<LOQ	<LOQ	3.2713 ± 0.3696
**Valeraldehyde** (0.619)				
Baseline	<LOQ	<LOQ	<LOQ	<LOQ
Pre-defined	<LOQ	<LOQ	n/a	n/a
Ad libitum	<LOQ	<LOQ	<LOQ	6.095 ± 0.887
**2,5-Dimethylbenzaldehyde** (0.884)				
Baseline	<LOQ	<LOQ	<LOQ	<LOQ
Pre-defined	<LOQ	<LOQ	n/a	n/a
Ad libitum	<LOQ	<LOQ	<LOQ	4.538 ± 0.843
**Methyl ethyl ketone** (1.45)				
Background before baseline	6.7150 ± 1.8311	2.2425 ± 2.7019	10.6700 ± 1.3361	9.6150 ± 1.1676
Baseline	5.1450 ± 0.7422	4.1088 ± 0.9380	6.9400 ± 1.1038	7.5450 ± 0.5572
Background before pre-defined	7.2200 ± 1.2561	4.4925 ± 0.6173	n/a	n/a
Pre-defined	7.1463 ± 1.9858	4.3075 ± 0.3774	n/a	n/a
Background before *ad libitum*	11.7650 ± 1.4732	5.4525 ± 2.2910	8.3300 ± 1.4816	7.5025 ± 0.7192
Ad libitum	9.0200 ± 0.8979	5.9000 ± 0.6691	7.4575 ± 0.9666	24.4750 ± 2.1991
**Acrolein** (0.619)				
Baseline	<LOQ	<LOQ	<LOQ	<LOQ
Pre-defined	<LOQ	<LOQ	n/a	n/a
Ad libitum	<LOQ	<LOQ	<LOQ	<LOQ
**1,3-Butadiene** (0.194)				
Baseline	<LOQ	<LOQ	<LOQ	<LOQ
Pre-defined	<LOQ	<LOQ	n/a	n/a
Ad libitum	<LOQ	<LOQ	<LOQ	10.5550 ± 0.5709
**Benzene** (0.281)				
Baseline	0.5843 ± 0.0280	1.4305 ± 0.1778	0.9145 ± 0.0474	2.2685 ± 0.0669
Pre-defined	0.8338 ± 0.0240	0.6823 ± 0.0075	n/a	n/a
Ad libitum	0.5400 ± 0.0039	0.7025 ± 0.1280	1.0605 ± 0.0152	15.5075 ± 0.4649
**Isoprene** (0.243)				
Baseline	7.4870 ± 0.4478	6.4588 ± 0.1464	7.5715 ± 0.1687	7.8865 ± 0.2324
Pre-defined	9.2058 ± 0.2934	7.0130 ± 0.2909	n/a	n/a
Ad libitum	7.0823 ± 1.8117	6.2833 ± 1.6497	9.4103 ± 0.2820	175.7000 ± 8.0403
**Toluene** (0.330)				
Baseline	1.0908 ± 0.2644	2.2078 ± 0.2517	2.0225 ± 0.4056	3.8565 ± 0.1952
Pre-defined	2.9933 ± 0.3542	0.1883 ± 0.2192	n/a	n/a
Ad libitum	1.5220 ± 0.2453	0.6803 ± 0.1808	2.1505 ± 0.0817	34.6825 ± 1.0908
**Furan** (0.178)				
Baseline	<LOQ	<LOQ	<LOQ	<LOQ
Pre-defined	<LOQ	<LOQ	n/a	n/a
Ad libitum	<LOQ	<LOQ	<LOQ	12.078 ± 0.497
**Ethylene oxide** (0.628)				
Baseline	<LOQ	<LOQ	<LOQ	<LOQ
Pre-defined	<LOQ	<LOQ	n/a	n/a

Ad libitum	<LOQ	<LOQ	<LOQ	<LOQ
**Vinyl chloride** (0.222)				
Baseline	<LOQ	<LOQ	<LOQ	<LOQ
Pre-defined	<LOQ	<LOQ	n/a	n/a
Ad libitum	<LOQ	<LOQ	<LOQ	0.1138 ± 0.2275
**Propylene oxide** (0.826)				
Baseline	<LOQ	<LOQ	<LOQ	<LOQ
Pre-defined	<LOQ	<LOQ	n/a	n/a
Ad libitum	<LOQ	<LOQ	<LOQ	<LOQ
**Nitromethane** (4.33)				
Baseline	<LOQ	<LOQ	<LOQ	<LOQ
Pre-defined	<LOQ	<LOQ	n/a	n/a
Ad libitum	<LOQ	<LOQ	<LOQ	<LOQ
**2-Nitropropane** (6.33)				
Baseline	<LOQ	<LOQ	<LOQ	<LOQ
Pre-defined	<LOQ	<LOQ	n/a	n/a
Ad libitum	<LOQ	<LOQ	<LOQ	<LOQ
**Vinyl acetate** (6.11)				
Baseline	<LOQ	<LOQ	<LOQ	<LOQ
Pre-defined	<LOQ	<LOQ	n/a	n/a
Ad libitum	<LOQ	<LOQ	<LOQ	<LOQ
**Ethylbenzene** (0.378)				
Baseline	<LOQ	<LOQ	<LOQ	1.0978 ± 0.0296
Pre-defined	<LOQ	<LOQ	n/a	n/a
Ad libitum	<LOQ	<LOQ	<LOQ	4.6200 ± 0.1589

Values are means of four samplers ± SD. Note: N: number of participants in the group; n/a: not applicable; NR: not reportable, compound was not quantifiable due to unknown matrix interferences;

<LOQ: below the level of quantification.

Table S2. Absolute levels of RAS constituents (µg/m3) measured during background (where detected), baseline and product use conditions, for all groups, for each of the four sampling port.

**Constituent (LOQ µg/m³)**	**Group I**	**Group II**	**Group III**	**Group IV**
Condition	(*N* = 9)	(*N* = 9)	(N=9)	(N=10)
Nicotine (0.248)				
Baseline	<LOQ, <LOQ, <LOQ, <LOQ	<LOQ, <LOQ, <LOQ, <LOQ	<LOQ, <LOQ, <LOQ, <LOQ	<LOQ, <LOQ, <LOQ, <LOQ
Pre-defined	0.294, 0.611, 0.386, 0.614	2.280, 3.350, 2.850, 2.830	n/a	n/a
Ad libitum	0.315, 0.376, 0.355, 0.478	0.992, 0.687, 0.929, 1.230	1.220, 1.190, 1.650, 1.830	34.700, 36.100, 43.500, 48.300
Propylene glycol (3.63)				
Baseline	17.60, 15.70, 15.70, 20.35	66.30, 57.40, 63.90, 65.35	59.10, 55.30, 63.60, 69.75	9.02, 8.43, 10.70, 10.90
Pre-defined	61.20, 63.95, 55.70, 67.95	257.00, 279.00, 278.00, 285.00	n/a	n/a
Ad libitum	47.90, 50.00, 48.70, 55.00	128.00, 129.00, 134.00, 136.00	361.00, 372.00, 396.00, 387.00	59.70, 64.30, 68.20, 71.70
Glycerol (4.11)				
Baseline	<LOQ, <LOQ, <LOQ, <LOQ	<LOQ, <LOQ, <LOQ, <LOQ	<LOQ, <LOQ, <LOQ, <LOQ	<LOQ, <LOQ, <LOQ, <LOQ
Pre-defined	62.90, 90.90, 50.80, 66.95	112.00, 143.00, 127.00, 125.00	n/a	n/a
Ad libitum	68.60, 136.00, 90.10, 100.90	70.20, 73.80, 80.50, 90.10	236.00, 250.00, 247.00, 235.00	<LOQ, <LOQ, <LOQ, <LOQ
Arsenic (0.123)				
Baseline	<LOQ, <LOQ, <LOQ, <LOQ	<LOQ, <LOQ, <LOQ, <LOQ	<LOQ, <LOQ, <LOQ, <LOQ	<LOQ, <LOQ, <LOQ, <LOQ
Pre-defined	<LOQ, <LOQ, <LOQ, <LOQ	<LOQ, <LOQ, <LOQ, <LOQ	n/a	n/a
Ad libitum	<LOQ, <LOQ, <LOQ, <LOQ	<LOQ, <LOQ, <LOQ, <LOQ	<LOQ, <LOQ, <LOQ, <LOQ	<LOQ, <LOQ, <LOQ, <LOQ
Cadmium (0.123)				
Baseline	<LOQ, <LOQ, <LOQ, <LOQ	<LOQ, <LOQ, <LOQ, <LOQ	<LOQ, <LOQ, <LOQ, <LOQ	<LOQ, <LOQ, <LOQ, <LOQ
Pre-defined	<LOQ, <LOQ, <LOQ, <LOQ	<LOQ, <LOQ, <LOQ, <LOQ	n/a	n/a
Ad libitum	<LOQ, <LOQ, <LOQ, <LOQ	<LOQ, <LOQ, <LOQ, <LOQ	<LOQ, <LOQ, <LOQ, <LOQ	<LOQ, <LOQ, <LOQ, <LOQ
Chromium (0.123)				
Baseline	0.124, <LOQ, <LOQ, 0.121	0.141, 0.136, 0.129, 0.142	0.133, 0.175, 0.173, <LOQ	0.133, 0.129, <LOQ, <LOQ
Pre-defined	<LOQ, 0.128, 0.138, <LOQ	<LOQ, <LOQ, <LOQ, <LOQ	n/a	n/a
Ad libitum	0.145, <LOQ, 0.146, <LOQ	<LOQ, <LOQ, <LOQ, <LOQ	0.126, 0.149, <LOQ, <LOQ	0.139, 0.146, 0.131, <LOQ
Nickel (0.123)				
Baseline	0.143, <LOQ, <LOQ, <LOQ	<LOQ, 0.285, 0.246, 0.288	<LOQ, <LOQ, <LOQ, <LOQ	<LOQ, <LOQ, <LOQ, <LOQ
Pre-defined	0.163, < LOQ, 0.13, 0.147	<LOQ, 0.125, <LOQ, <LOQ	n/a	n/a
Ad libitum	<LOQ, <LOQ, <LOQ, <LOQ	<LOQ, <LOQ, <LOQ, <LOQ	<LOQ, <LOQ, <LOQ, <LOQ	<LOQ, <LOQ, <LOQ, <LOQ
Formaldehyde (0.619)				
Background before baseline	4.01, 1.96, 2.63, 2.36	<LOQ, <LOQ, 2.90, <LOQ	2.18, 2.48, 2.76, 2.99	2.90, 2.37, 1.95, 2.82
Baseline	3.59, 3.50, 3.55, 4.12	5.84, 4.27, 8.04, 4.59	3.49, 3.67, 3.18, 3.39	4.33, 4.28, 4.53, 4.36
Background before pre-defined	5.25, 8.86, 5.51, 4.55	2.92, 4.47, 4.08, 4.41	n/a	n/a
Pre-defined	5.36, 5.26, 5.45, 5.35	4.75, 5.42, 5.05, 4.67	n/a	n/a
Background before *ad libitum*	10.10, 14.80, 13.20, 9.08	4.27, 5.97, 7.60, 7.11	4.13, 3.49, 3.52, 3.77	3.58, 3.57, 3.43, 3.42
Ad libitum	9.78, 8.08, 8.58, 10.20	5.98, 5.82, 5.81, 5.68	3.94, 3.14, 4.24, 4.19	50.00, 55.80, 54.60, 60.00

Crotonaldehyde (0.619)				
Baseline	<LOQ, <LOQ, <LOQ, <LOQ	<LOQ, <LOQ, <LOQ, <LOQ	<LOQ, <LOQ, <LOQ, <LOQ	<LOQ, <LOQ, <LOQ, <LOQ
Pre-defined	<LOQ, <LOQ, <LOQ, <LOQ	<LOQ, <LOQ, <LOQ, <LOQ	n/a	n/a
Ad libitum	<LOQ, <LOQ, <LOQ, <LOQ	<LOQ, <LOQ, <LOQ, <LOQ	<LOQ, <LOQ, <LOQ, <LOQ	1.315, 1.199, <LOQ, 1.860
o-Tolualdehyde (0.0149)				
Baseline	<LOQ, <LOQ, <LOQ, <LOQ	<LOQ, <LOQ, <LOQ, <LOQ	<LOQ, <LOQ, <LOQ, <LOQ	<LOQ, <LOQ, <LOQ, <LOQ
Pre-defined	<LOQ, <LOQ, <LOQ, <LOQ	<LOQ, <LOQ, <LOQ, <LOQ	n/a	n/a
Ad libitum	<LOQ, <LOQ, <LOQ, <LOQ	<LOQ, <LOQ, <LOQ, <LOQ	<LOQ, <LOQ, <LOQ, <LOQ	<LOQ, <LOQ, <LOQ, <LOQ
Acetaldehyde (0.619)				
Background before baseline	3.940, 2.270, 2.760, 3.080	<LOQ, <LOQ, 2.580, <LOQ	4.340, 5.440, 5.070, 4.610	4.480, 4.620, 4.890, 4.660
Baseline	3.585, 3.400, 3.165, 3.705	2.830, 2.675, 3.390, 2.295	4.030, 3.870, 3.570, 3.840	3.850, 4.220, 4.120, 4.220
Background before pre-defined	3.820, 7.910, 5.580, 3.810	2.930, 3.570, 4.070, 4.120	n/a	n/a
Pre-defined	4.030, 3.630, 3.745, 4.320	3.390, 3.670, 4.195, 3.455	n/a	n/a
Background before *ad libitum*	8.510, 8.450, 10.500, 7.480	4.640, 5.430, 5.660, 6.170	4.040, 4.270, 4.030, 4.800	5.050, 6.130, 4.740, 5.440
Ad libitum	6.585, 5.745, 6.085, 7.215	4.560, 5.995, 6.070, 14.290	5.210, 4.890, 5.810, 5.620	104.000, 110.000, 111.000, 117.000
Butyraldehyde (0.698)				
Baseline	<LOQ, <LOQ, <LOQ, <LOQ	<LOQ, <LOQ, <LOQ, <LOQ	<LOQ, <LOQ, <LOQ, <LOQ	<LOQ, <LOQ, <LOQ, <LOQ
Pre-defined	<LOQ, <LOQ, <LOQ, <LOQ	<LOQ, <LOQ, <LOQ, <LOQ	n/a	n/a
Ad libitum	<LOQ, <LOQ, <LOQ, <LOQ	<LOQ, <LOQ, <LOQ, <LOQ	<LOQ, <LOQ, <LOQ, <LOQ	5.45, 5.70, 7.37, 5.95
m- and p-Tolualdehyde (1.240)				
Baseline	<LOQ, <LOQ, <LOQ, <LOQ	<LOQ, <LOQ, <LOQ, <LOQ	<LOQ, <LOQ, <LOQ, <LOQ	<LOQ, <LOQ, <LOQ, <LOQ
Pre-defined	<LOQ, <LOQ, <LOQ, <LOQ	<LOQ, <LOQ, <LOQ, <LOQ	n/a	n/a
Ad libitum	<LOQ, <LOQ, <LOQ, <LOQ	<LOQ, <LOQ, <LOQ, <LOQ	<LOQ, <LOQ, <LOQ, <LOQ	<LOQ, <LOQ, <LOQ, <LOQ
Acetone (0.619)				
Background before baseline	65.30, 9.57, 0.00, 11.60	<LOQ, <LOQ, 15.75, <LOQ	NR, NR, NR, NR	31.80, 27.60, 34.10, 14.70
Baseline	16.10, 14.95, 16.10, 16.90	23.00, 20.30, 26.65, 14.05	NR, NR, NR, NR	35.40, 45.30, 42.30, 40.70
Background before pre-defined	23.10, 38.40, 64.30, 16.20	8.87, 14.20, 14.70, 22.00	n/a	n/a
Pre-defined	25.15, 26.15, 26.10, 26.05	28.65, 33.30, 35.60, 26.65	n/a	n/a
Background before *ad libitum*	36.40, 33.10, 67.40, 28.50	11.80, 17.90, 20.70, 36.10	NR, NR, NR, NR	19.70, 11.30, 23.70, 21.40
Ad libitum	42.35, 45.10, 49.95, 44.95	28.05, 28.00, 27.15, 37.75	NR, NR, NR, NR	71.40, 82.70, 75.90, 84.40
Benzaldehyde (0.619)				
Baseline	<LOQ, <LOQ, <LOQ, <LOQ	<LOQ, <LOQ, <LOQ, <LOQ	<LOQ, <LOQ, <LOQ, <LOQ	<LOQ, <LOQ, <LOQ, <LOQ
Pre-defined	<LOQ, <LOQ, <LOQ, <LOQ	<LOQ, <LOQ, <LOQ, <LOQ	n/a	n/a
Ad libitum	<LOQ, <LOQ, <LOQ, <LOQ	<LOQ, <LOQ, <LOQ, <LOQ	<LOQ, <LOQ, <LOQ, <LOQ	<LOQ, <LOQ, <LOQ, <LOQ, 2.12
Propionaldehyde (0.798)				
Baseline	<LOQ, <LOQ, <LOQ, <LOQ	NR, <LOQ, NR, <LOQ	<LOQ, <LOQ, <LOQ, <LOQ	<LOQ, <LOQ, <LOQ, <LOQ
Pre-defined	<LOQ, <LOQ, <LOQ, <LOQ	<LOQ, NR, <LOQ, <LOQ	n/a	n/a
Ad libitum	NR, NR, NR, NR	<LOQ, <LOQ, <LOQ, <LOQ	<LOQ, <LOQ, <LOQ, <LOQ	<LOQ, <LOQ, <LOQ, <LOQ
Isovaleraldehyde (0.619)				

Baseline	<LOQ, <LOQ, <LOQ, <LOQ	<LOQ, <LOQ, <LOQ, <LOQ	<LOQ, <LOQ, <LOQ, <LOQ	<LOQ, <LOQ, <LOQ, <LOQ
Pre-defined	<LOQ, <LOQ, <LOQ, <LOQ	<LOQ, <LOQ, <LOQ, <LOQ	n/a	n/a
Ad libitum	<LOQ, <LOQ, <LOQ, <LOQ	<LOQ, <LOQ, <LOQ, <LOQ	<LOQ, <LOQ, <LOQ, <LOQ	2.12, 2.18, 1.35, 2.73
Hexaldehyde (1.200)				
Baseline	<LOQ, <LOQ, <LOQ, <LOQ	<LOQ, <LOQ, <LOQ, <LOQ	<LOQ, <LOQ, <LOQ, <LOQ	<LOQ, <LOQ, <LOQ, <LOQ
Pre-defined	<LOQ, <LOQ, <LOQ, <LOQ	<LOQ, <LOQ, <LOQ, <LOQ	n/a	n/a
Ad libitum	1.909, 2.667, 1.440, 2.263	<LOQ, <LOQ, <LOQ, <LOQ	<LOQ, <LOQ, <LOQ, <LOQ	3.485, 2.890, 3.035, 3.675
Valeraldehyde (0.619)				
Baseline	<LOQ, <LOQ, <LOQ, <LOQ	<LOQ, <LOQ, <LOQ, <LOQ	<LOQ, <LOQ, <LOQ, <LOQ	<LOQ, <LOQ, <LOQ, <LOQ
Pre-defined	<LOQ, <LOQ, <LOQ, <LOQ	<LOQ, <LOQ, <LOQ, <LOQ	n/a	n/a
Ad libitum	<LOQ, <LOQ, <LOQ, <LOQ	<LOQ, <LOQ, <LOQ, <LOQ	<LOQ, <LOQ, <LOQ, <LOQ	6.24, 5.50, 5.35, 7.29
2,5-Dimethylbenzaldehyde (0.884)				
Baseline	<LOQ, <LOQ, <LOQ, <LOQ	<LOQ, <LOQ, <LOQ, <LOQ	<LOQ, <LOQ, <LOQ, <LOQ	<LOQ, <LOQ, <LOQ, <LOQ
Pre-defined	<LOQ, <LOQ, <LOQ, <LOQ	<LOQ, <LOQ, <LOQ, <LOQ	n/a	n/a
Ad libitum	<LOQ, <LOQ, <LOQ, <LOQ	<LOQ, <LOQ, <LOQ, <LOQ	<LOQ, <LOQ, <LOQ, <LOQ	5.46, 3.66, 4.01, 5.02
Methyl ethyl ketone (1.45)				
Background before baseline	9.040, 4.610, 6.920, 6.290	<LOQ, 3.540, 5.430, <LOQ	9.830, 11.600, 12.000, 9.250	8.190, 11.000, 9.900, 9.370
Baseline	6.040, 4.255, 4.970, 5.315	4.195, 4.150, 5.190, 2.900	7.070, 5.900, 6.360, 8.430	6.790, 7.550, 8.120, 7.720
Background before pre-defined	7.640, 8.700, 6.800, 5.740	3.630, 5.020, 4.480, 4.840	n/a	n/a
Pre-defined	10.115, 6.385, 6.055, 6.030	4.435, 4.520, 4.530, 3.745	n/a	n/a
Background before *ad libitum*	11.200, 12.700, 13.200, 9.960	3.700, 4.770, 4.520, 8.820	7.890, 7.770, 7.160, 10.500	6.840, 7.740, 8.410, 7.020
Ad libitum	9.235, 7.885, 8.905, 10.055	5.205, 5.450, 6.505, 6.440	7.370, 6.270, 7.560, 8.630	21.200, 25.200, 25.800, 25.700
Acrolein (0.619)				
Baseline	<LOQ, <LOQ, <LOQ, <LOQ	<LOQ, <LOQ, <LOQ, <LOQ	<LOQ, <LOQ, <LOQ, <LOQ	<LOQ, <LOQ, <LOQ, <LOQ
Pre-defined	<LOQ, <LOQ, <LOQ, <LOQ	<LOQ, <LOQ, <LOQ, <LOQ	n/a	n/a
Ad libitum	<LOQ, <LOQ, <LOQ, <LOQ	<LOQ, <LOQ, <LOQ, <LOQ	<LOQ, <LOQ, <LOQ, <LOQ	<LOQ, <LOQ, <LOQ, <LOQ
1,3-Butadiene (0.194)				
Baseline	<LOQ, <LOQ, <LOQ, <LOQ	<LOQ, <LOQ, <LOQ, <LOQ	<LOQ, <LOQ, <LOQ, <LOQ	<LOQ, <LOQ, <LOQ, <LOQ

Pre-defined	<LOQ, <LOQ, <LOQ, <LOQ	<LOQ, <LOQ, <LOQ, <LOQ	n/a	n/a
Ad libitum	<LOQ, <LOQ, <LOQ, <LOQ	<LOQ, <LOQ, <LOQ, <LOQ	<LOQ, <LOQ, <LOQ, <LOQ	9.870, 10.310, 10.930, 11.110
Benzene (0.281)				
Baseline	0.586, 0.596, 0.545, 0.610	1.587, 1.582, 1.275, 1.278	0.975, 0.863, 0.924, 0.896	2.250, 2.273, 2.356, 2.195
Pre-defined	0.846, 0.860, 0.806, 0.823	0.678, 0.692, 0.675, 0.684	n/a	n/a
Ad libitum	0.538, 0.541, 0.536, 0.545	0.770, 0.775, 0.754, 0.511	1.059, 1.075, 1.068, 1.040	14.910, 15.370, 15.840, 15.910
Isoprene (0.243)				
Baseline	7.376, 7.118, 7.316, 8.138	6.373, 6.325, 6.482, 6.655	7.591, 7.346, 7.594, 7.755	7.633, 7.821, 7.900, 8.192
Pre-defined	8.861, 9.109, 9.299, 9.554	6.932, 6.672, 7.079, 7.369	n/a	n/a
Ad libitum	7.657, 7.929, 4.398, 8.345	7.027, 6.918, 7.363, 3.825	9.215, 9.160, 9.494, 9.772	166.400, 173.700, 176.900, 185.800
Toluene (0.330)				
Baseline	0.948, 1.296, 0.790, 1.329	2.390, 2.391, 1.857, 2.193	2.603, 1.658, 1.928, 1.901	4.045, 3.659, 4.002, 3.720
Pre-defined	3.079, 3.456, 2.766, 2.672	0.411, <LOQ, <LOQ, 0.342	n/a	n/a
Ad libitum	1.720, 1.614, 1.164, 1.590	0.662, 0.854, 0.769, 0.436	2.109, 2.273, 2.107, 2.113	33.260, 34.490, 35.170, 35.810
Furan (0.178)				
Baseline	<LOQ, <LOQ, <LOQ, <LOQ	<LOQ, <LOQ, <LOQ, <LOQ	<LOQ, <LOQ, <LOQ, <LOQ	<LOQ, <LOQ, <LOQ, <LOQ
Pre-defined	<LOQ, <LOQ, <LOQ, <LOQ	<LOQ, <LOQ, <LOQ, <LOQ	n/a	n/a
Ad libitum	<LOQ, <LOQ, <LOQ, <LOQ	<LOQ, <LOQ, <LOQ, <LOQ	<LOQ, <LOQ, <LOQ, <LOQ	11.50, 11.99, 12.11, 12.71
Ethylene oxide (0.628)				
Baseline	<LOQ, <LOQ, <LOQ, <LOQ	<LOQ, <LOQ, <LOQ, <LOQ	<LOQ, <LOQ, <LOQ, <LOQ	<LOQ, <LOQ, <LOQ, <LOQ
Pre-defined	<LOQ, <LOQ, <LOQ, <LOQ	<LOQ, <LOQ, <LOQ, <LOQ	n/a	n/a
Ad libitum	<LOQ, <LOQ, <LOQ, <LOQ	<LOQ, <LOQ, <LOQ, <LOQ	<LOQ, <LOQ, <LOQ, <LOQ	<LOQ, <LOQ, <LOQ, <LOQ
Vinyl chloride (0.222)				
Baseline	<LOQ, <LOQ, <LOQ, <LOQ	<LOQ, <LOQ, <LOQ, <LOQ	<LOQ, <LOQ, <LOQ, <LOQ	<LOQ, <LOQ, <LOQ, <LOQ
Pre-defined	<LOQ, <LOQ, <LOQ, <LOQ	<LOQ, <LOQ, <LOQ, <LOQ	n/a	n/a
Ad libitum	<LOQ, <LOQ, <LOQ, <LOQ	<LOQ, <LOQ, <LOQ, <LOQ	<LOQ, <LOQ, <LOQ, <LOQ	<LOQ, <LOQ, <LOQ, 0.455
Propylene oxide (0.826)				
Baseline	<LOQ, <LOQ, <LOQ, <LOQ	<LOQ, <LOQ, <LOQ, <LOQ	<LOQ, <LOQ, <LOQ, <LOQ	<LOQ, <LOQ, <LOQ, <LOQ

Pre-defined	<LOQ, <LOQ, <LOQ, <LOQ	<LOQ, <LOQ, <LOQ, <LOQ	n/a	n/a
Ad libitum	<LOQ, <LOQ, <LOQ, <LOQ	<LOQ, <LOQ, <LOQ, <LOQ	<LOQ, <LOQ, <LOQ, <LOQ	<LOQ, <LOQ, <LOQ, <LOQ
Nitromethane (4.33)				
Baseline	<LOQ, <LOQ, <LOQ, <LOQ	<LOQ, <LOQ, <LOQ, <LOQ	<LOQ, <LOQ, <LOQ, <LOQ	<LOQ, <LOQ, <LOQ, <LOQ
Pre-defined	<LOQ, <LOQ, <LOQ, <LOQ	<LOQ, <LOQ, <LOQ, <LOQ	n/a	n/a
Ad libitum	<LOQ, <LOQ, <LOQ, <LOQ	<LOQ, <LOQ, <LOQ, <LOQ	<LOQ, <LOQ, <LOQ, <LOQ	<LOQ, <LOQ, <LOQ, <LOQ
2-Nitropropane (6.33)				
Baseline	<LOQ, <LOQ, <LOQ, <LOQ	<LOQ, <LOQ, <LOQ, <LOQ	<LOQ, <LOQ, <LOQ, <LOQ	<LOQ, <LOQ, <LOQ, <LOQ
Pre-defined	<LOQ, <LOQ, <LOQ, <LOQ	<LOQ, <LOQ, <LOQ, <LOQ	n/a	n/a
Ad libitum	<LOQ, <LOQ, <LOQ, <LOQ	<LOQ, <LOQ, <LOQ, <LOQ	<LOQ, <LOQ, <LOQ, <LOQ	<LOQ, <LOQ, <LOQ, <LOQ
Vinyl acetate (6.11)				
Baseline	<LOQ, <LOQ, <LOQ, <LOQ	<LOQ, <LOQ, <LOQ, <LOQ	<LOQ, <LOQ, <LOQ, <LOQ	<LOQ, <LOQ, <LOQ, <LOQ
Pre-defined	<LOQ, <LOQ, <LOQ, <LOQ	<LOQ, <LOQ, <LOQ, <LOQ	n/a	n/a
Ad libitum	<LOQ, <LOQ, <LOQ, <LOQ	<LOQ, <LOQ, <LOQ, <LOQ	<LOQ, <LOQ, <LOQ, <LOQ	<LOQ, <LOQ, <LOQ, <LOQ
Ethylbenzene (0.378)				
Baseline	<LOQ, <LOQ, <LOQ, <LOQ	<LOQ, <LOQ, <LOQ, <LOQ	<LOQ, <LOQ, <LOQ, <LOQ	1.096, 1.074, 1.140, 1.081
Pre-defined	<LOQ, <LOQ, <LOQ, <LOQ	<LOQ, <LOQ, <LOQ, <LOQ	n/a	n/a
Ad libitum	<LOQ, <LOQ, <LOQ, <LOQ	<LOQ, <LOQ, <LOQ, <LOQ	<LOQ, <LOQ, <LOQ, <LOQ	4.421, 4.566, 4.722, 4.771

Note: N: number of participants in the group; n/a: not applicable; NR: not reportable, compound was not quantifiable due to unknown matrix interferences; <LOQ: below the level of quantification.

Table S3. Absolute levels of nicotine, propylene glycol and glycerol (µg/cm2) in each surface sample location during each product use condition

**Constituent (LOQ µg/cm²)**	**Group I**				**Group II**				**Group III**				**Group IV**			
	(N=9)				(N=9)				(N=9)				(N=10)			
Condition	Sample 1	Sample 2	Sample 3	Sample 4	Sample 1	Sample 2	Sample 3	Sample 4	Sample 1	Sample 2	Sample 3	Sample 4	Sample 1	Sample 2	Sample 3	Sample 4
**Nicotine** (0.00126)																

Baseline	<LOQ	<LOQ	<LOQ	<LOQ	<LOQ	<LOQ	<LOQ	<LOQ	<LOQ	<LOQ	<LOQ	<LOQ	<LOQ	<LOQ	<LOQ	<LOQ
Pre-defined	0.00308	<LOQ	<LOQ	<LOQ	<LOQ	<LOQ	<LOQ	<LOQ	n/a	n/a	n/a	n/a	n/a	n/a	n/a	n/a
Ad libitum	<LOQ	<LOQ	<LOQ	<LOQ	<LOQ	<LOQ	<LOQ	<LOQ	<LOQ	<LOQ	<LOQ	<LOQ	<LOQ	0.00437	<LOQ	<LOQ
**Propylene glycol** (0.0185)																
Baseline	<LOQ	<LOQ	<LOQ	<LOQ	<LOQ	<LOQ	<LOQ	<LOQ	<LOQ	<LOQ	<LOQ	<LOQ	<LOQ	<LOQ	<LOQ	<LOQ
Pre-defined	0.0249	0.0189	0.0201	<LOQ	<LOQ	<LOQ	0.0194	<LOQ	n/a	n/a	n/a	n/a	n/a	n/a	n/a	n/a
Ad libitum	<LOQ	<LOQ	<LOQ	<LOQ	0.0262	0.0253	<LOQ	<LOQ	<LOQ	<LOQ	<LOQ	<LOQ	<LOQ	<LOQ	<LOQ	<LOQ
**Glycerol** (0.0209)																
Baseline	<LOQ	<LOQ	<LOQ	<LOQ	<LOQ	<LOQ	<LOQ	<LOQ	<LOQ	<LOQ	<LOQ	<LOQ	<LOQ	<LOQ	<LOQ	<LOQ
Pre-defined	<LOQ	<LOQ	<LOQ	<LOQ	0.0819	0.0829	0.078	0.0321	n/a	n/a	n/a	n/a	n/a	n/a	n/a	n/a
Ad libitum	0.025	<LOQ	0.0258	0.0281	<LOQ	<LOQ	<LOQ	<LOQ	0.2701	0.511	0.325	0.2395	<LOQ	<LOQ	<LOQ	<LOQ

Note: N: number of participants in the group; n/a: not applicable; LOQ: limit of quantification.
